# Psychological and socio-cultural factors affecting latrine ownership and usage in rural South India

**DOI:** 10.6026/973206300200502

**Published:** 2024-05-31

**Authors:** Sebastian Nancy, Kamal Batcha Mujibur Rahman, Selvaraju Sathish Kumar, Kasinathan Murugesan, Uthirapathy Udhayakumar

**Affiliations:** 1 Department of Community Medicine, Vinayaka Mission's Medical College and Hospital, Vinayaka Mission's Research Foundation - Deemed to be University (VMRF-DU), Karaikal, Puducherry, India

**Keywords:** latrine, ownership, utilization, psycho-social, sanitation

## Abstract

The levels of latrine utilization were lower than the levels of latrine ownership in rural areas owing to certain psycho-social barriers hindering latrine construction and consistent utilization. The study was aimed to identify the proportion of latrine construction and usage and to explore the psychological, socio-cultural and structural factors influencing latrine ownership and utilization. A community-based cross-sectional study was carried out at the four villages of Villupuram district for three months. After IEC clearance, information was collected from a representative sample of 422 households. Direct observation of the latrines was employed along with surveys. The data were entered and analysed in MS Excel. Categorical variables were expressed as frequencies and percentages. Further, the reasons were categorized into psychological, socio-cultural and structural factors. About 54.7% households owned a latrine and among them 46.8% were using it. Psycho-social factors such as convenience, habitual nature, privacy and space constraints in open defecation influenced latrine ownership. Fear of snakes and insects, safety and protection, time saving and proper functioning latrines with availability of water facilitated latrine utilization are of concern. A multi-faceted strategy employing context-specific behaviour change communication along with Government's financial support would improve both latrine construction and utilization.

## Background:

The negative public health impact of open defecation could be neutralized by latrine adoption. Latrine usage prevented diarrhoea, polio, and cholera and hookworm infestation [1]. Improved sanitation could prevent malnutrition and under-five deaths [1]. Besides, latrine usage promoted dignity and status of females [2]. Better sanitation ensured potential recovery of water and renewable energy and nutrients from faecal waste [2]. Additionally, improved sanitation lowered health costs, increased productivity and reduced premature deaths [3]. According to World Health Organization (WHO), globally an estimated 2.3 billion people lacked access to basic sanitation facilities and 892 million people defecated in the open [3]. In India, fourth round of National Family Health Survey (NFHS-4) in 2015 to 2016 found that about 39% of the Indian households were practicing open defecation and less than half 48% households were using a non-shared improved latrine facility [4]. As per NFHS-4 report, in Tamil Nadu, overall 52% of households do not use an improved sanitation facility and open defecation was more prevalent in rural households (61%) than in urban households (17%) [4]. Notably, psycho-social factors like safety, hygiene, disease prevention and prestige were the primary facilitators in latrine possession while lack of space, money and comfort in open defecation were the hindrances in latrine possession in rural areas [5]. Likewise, the factors which facilitated latrine usage were having a close/ private latrine and access to enough water [6]. While factors which hindered latrine usage were unavailability of water, expensive soap and space constraints [6]. In addition, the positive consequences reported in latrine usage were snake bite prevention, cleanliness, time saving, comfortable in night and rainy season and safe from harm. Whereas, the negative consequences reported in latrine utilization were too much consumption of water and frequent cleaning [6]. Therefore, it is of interest to identify the proportion of latrine construction and usage and to explore the psychological, socio-cultural and structural factors influencing latrine ownership and utilization in resource poor settings.

## Methodology:

A community-based cross-sectional study was executed in four villages of Villupuram district namely, Ayyur, Agaram, Pidagam, Kappur and Anangoor. These villages belonged to Vikkiravandi and Kolianur blocks of Villupuram district and covered 4,409 households. The study was conducted for a period of three months (October 2020 to December 2020) which was the time needed for data collection and analysis. Considering 50% of the households were using improved sanitation facility as per NFHS-4 (2015-16) data [4], a representative sample of 383 was calculated using OpenEpi software (version 3.01) with 95% confidence interval, 5% absolute precision and 80% power. Considering 10% non-response rate, the final sample size was 422 households. At first, households in the sampling frame were assigned with a number. Then, 422 sample households were selected from the sampling frame by Simple Random Sampling (SRS) using random numbers generated through Open Epi software (version 3.01). The unit of the study was the households. Female head of the family was interviewed with the assumption that she would know the reasons for the behaviour of her family members. In order to ensure autonomy, only the respondents who gave consent for both the interview and observation of the latrine were included. After an initial brief introduction, the principal investigator obtained a written informed consent for both the interview and observation from the female head of the family. We used a pre-tested structured questionnaire to collect information about the latrine facility and usage. Direct observation of the latrines was employed along with surveys in order to confirm the self-reported latrine ownership. The information regarding the psycho-social facilitators in latrine ownership and usage was collected from the latrine owners and users. The collected data were entered and analysed in MS Excel. Categorical variables were expressed as frequencies and percentages. In addition, the reasons given for latrine construction and usage were categorized into psychological, socio-cultural and structural factors.

## Ethical consideration:

The study was carried out after obtaining approval from the Research Committee and Institutional Ethics Committee (EC approval number: 40/2020).

## Results:

## Socio-demographic characteristics of the households in the study villages:

Majority of the female respondents, 382 (90.5%) were between the age group of 20 - 60 years and only 40 (9.5%) were above 60 years. Among 422 households interviewed, nearly half 198 (46.9%) of the head of the family were unskilled workers followed by 116 (27.5%) skilled or semi-skilled workers, 44 (10.4%) clerks, shop-owners or farm-owners, 40 (9.5%) housewives, 21 (5%) retired or unemployed persons and three (0.7%) professionals or semi-professionals. About 138 (32.7%) head of the family were uneducated and among the educated group, half of them 210 (49.8%) received primary education, 53 (12.6%) completed higher secondary and only 21 (5%) were graduates. Majority of the households, 417 (98.8%) practiced Hinduism and only five (1.2%) households followed other religions like Christianity and Islam. Almost 299 (70.8%) households belonged to scheduled castes and only 123 (29.1%) belonged to backward castes. About 158 (37.4%), 153 (36.3%) and 111 (26.3%) households live in a pucca, semi-pucca and kutcha houses respectively. A majority of the households, 299 (70.8%) hail from a nuclear family, 67 (15.9%) households live in a joint family system and 56 (13.3%) households belong to a three-generation family. Notably 340 (80.6%) households had only one to five members in the family, 79 (18.7%) households had six to ten family members and only about three (0.7%) households had more than ten family members. More than half of the households, 258 (61.1%) were above poverty line while 164 (38.9%) households were below poverty line. (Table 1)

## Proportion of latrine ownership and utilization in the study villages:

**Figure 1:**
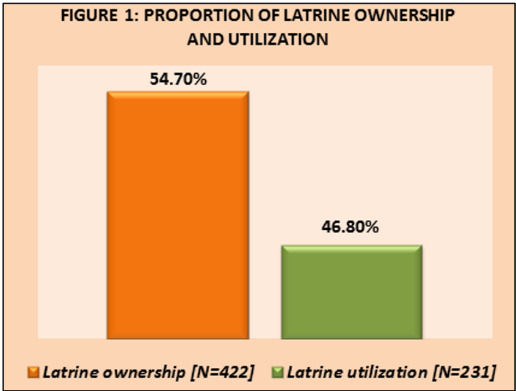
Proportion of latrine ownership and utilization in the study villages

Among 422 sample households, more than half 231 (54.7%) owned an individual household latrine. Out of 231 households who owned a latrine, almost 108 (46.8%) were using the latrine. (Figure 1)

## Households' self-perceived reasons for owning a latrine:

Among the psychological factors influencing latrine ownership, nearly 69 (29.9%) households in stated that having a latrine was convenient. About nine (3.9%) households had a latrine because it was habitual. Out of 231 households who had a latrine, about 43 (18.6%) and 47 (20.3%) respondents mentioned reasons such as privacy and safety respectively. Notably, 8 (3.5%) households reported that latrine maintained cleanliness and hygiene. About 13 (5.6%) respondents revealed that possessing a latrine in the house prevented diseases and snake bites. Nearly 39 (16.9%) households in the end-line survey reported that they had a latrine only for emergency purposes. About nine (3.9%) households were ignorant about the purpose of latrine. Dignity and status were one of the socio-cultural factors manipulating latrine possession in 45 (19.5%) households. About six (2.6%) households considered that latrine in the house were only for the usage of women, adolescent girls and guests. Among the structural factors determining latrine ownership, reduced space for open defecation was one the reason given by three (1.3%) respondents. About 19 (8.2%) respondents expressed that they had a latrine in their house because it was constructed by the government for free. (Table 2)

## Households' self-perceived reasons for using the owned latrine:

Among the psychological factors, convenience and privacy in latrine usage was proposed by 34 (31.5%) and 31 (28.7%) households respectively. About 34 (31.5%) households used a latrine because it prevented snake bites and diseases. Latrine provided safety and protection to nine (8.3%) households. Nearly 14 (13%) respondents expressed that latrine usage saved time. Notably 18 (16.7%) respondents considered that open defecation was disgusting. About two (1.8%) households revealed that latrine was used only for emergency purposes. Dignity and status was one of the socio-cultural factors influencing latrine usage in 21 (19.4%) households. Notably 13 (12%) households considered that latrine usage was related to education and occupation. Almost 64 (59.3%) households reported that proper functioning latrine was one the primary structural factor for using the owned latrine. About 52 (48.1%) respondents used a latrine due to availability of water source nearby and nearly 38 (35.2%) respondents used a latrine as it was built near their house. The remaining 26 (24.1%) respondents reported that adequate lighting inside the latrine facilitated latrine utilization. Interestingly, 10 (9.3%) households revealed that latrine usage produces good manure for the crops. (Table 3)

## Discussion:

In the present study, about 54.7% households owned an individual household latrine and among them 46.8% were using the owned latrine consistently. Psycho-social factors such as convenience, habitual nature, privacy, safety, cleanliness, dignity and status and space constraints in open defecation positively impacted latrine ownership. Whereas, fear of snakes and insects, safety and protection, time saving, emergency usage, dignity and status, education and occupation and proper functioning latrines with availability of water source facilitated latrine utilization. Sheethal MP *et al* carried out a cross-sectional study and found that latrine was present in about 82% of houses but more than half of the latrines lacked water facilities [5]. Only 18% practiced open defecation [5]. The study findings revealed that safety, hygiene, disease prevention and prestige were the primary facilitators in latrine possession while lack of space, money and comfort in open defecation were the hindrances in latrine possession [5]. In Kuthambakkam village, Tamil Nadu a community-based cross-sectional study found that the prevalence of usage of household sanitary latrine and community latrines were 62.5% and 4.3% respectively [7]. The prevalence of open defecation was 33.1% and a substantial association was found between low standard of living and open defecation [7]. Another cross-sectional study in Northern Karnataka showed that about 59.5% households had a latrine but only 77.3% of them were using it consistently [8]. Notably, this study showed that only 66% households were aware about SBM campaigns [8]. Lack of space was the common reason stated for not owning a latrine while socio-economic status, educational status and foot ware usage habit were associated with latrine ownership [8]. A community-based cross-sectional study in rural Ethiopia by Alemu *et al.* identified that 73% owned a latrine and among them 79% were using it consistently [9]. Among the psychological factors, attitude and injunctive norm predicted latrine ownership [9]. Further, large family size, education, having a school going child in the family and participation in Community Led Total Sanitation and Hygiene were associated with latrine ownership whereas having a clean and protected latrine was associated with consistent latrine use [9]. Higher proportion of villagers reported that having a latrine was convenient. Similarly, in six states of India, a barrier analysis study found that higher percentage of latrine users proposed that latrine was convenient as it saves time compared to latrine non-users [6]. Routray *et al.* in an exploratory qualitative study in Orissa also found that convenience was the primary reason for latrine construction [10]. Thus, people gradually change their negative attitude about latrines if they get accustomed to latrine usage.

Some households revealed that latrine was used only by the females in the house for their safety and protection. Males refused to use a latrine owing to misconceptions such as convenience in open defecation but allowed the females in the house to use it for their safety and protection. In a cross-sectional study in Karnataka, majority of the study subjects considered a latrine only for the safety of females and children [5]. Similarly, in a process evaluation study in Orissa mobilization activities like wall paintings, school rallies and follow up door-to-door household visits significantly reduced the percentage of households who narrated safety for married women as the key reason for latrine usage [11]. Hence, frequent reinforcement of key messages through multiple activities successfully changes the behaviours of hostile target audiences. The villagers failed to use a latrine owing to unawareness. A community-based cross-sectional study in Tamil Nadu found that latrine non-utilization was on account of unawareness [7]. In a longitudinal study in Orissa, almost 80% of the households preferred open defecation despite having a latrine owing to lack of awareness [12]. An exploratory qualitative study by Routray *et al.*. in Orissa revealed that latrine was adopted only by people who had exposure to them and understood their advantages [10]. Evaluation of TSC in Orissa showed that the levels of awareness about latrines were lower than the levels of latrine coverage indicating a need to accelerate mobilization activities [11]. Hence, ignorance about latrines played a key role in latrine non-utilization and various sensitization activities were used to create awareness among rural masses. We found that dignity and status determined latrine ownership. Here, the word 'dignity' signifies the right of women to be valued, respected and treated ethically. In India, a cross-sectional study carried out in five states showed that dignity and status played a major role in latrine construction which was similar to our present study [13]. Likewise, in Orissa, a community-based study found that IEC activities and adolescent girl's group formation significantly increased the percentage of households who gave reasons such as dignity and status for possessing a latrine [11]. Therefore, community-based women groups played a major role in addressing dignity and status in latrine ownership.

Structural factors such as reduced space for open defecation influenced latrine construction. Some villagers had an attitude that latrine was just an alternative to open defecation and they lacked awareness regarding the health benefits of latrine usage. Similar to our study findings, in Orissa, an impact evaluation of TSC programme which exhibited more thrust on school sanitation along with financial assistance marginally reduced the structural reasons given for latrine possession such as space constraints for open defecation [12]. Therefore, younger ones in the community helped people to understand the benefits of latrine adoption through various sensitization activities. Notably, a few households voiced out that latrine utilization was related to better socioeconomic status such as education and occupation. Households considered that latrine was a luxury item and should be used only by people with better education and occupation. A study done by Surya *et al.*. in five states of India showed that open defecation was significantly associated with lower socioeconomic status owing to inability to construct and maintain latrines constrained by poverty [13]. Thus, socioeconomic barriers hindering latrine usage were effectively tackled by positive deviants approach and through cultural exchange from urban to rural areas.

In the present study, latrine infrastructure and its' proper functioning positively influenced consistent latrine utilization in resource poor settings. A barrier analysis survey in six states of India showed that private latrine ownership, availability of water and other hygiene materials were related to latrine usage [6]. In rural Mali, CLTS interventions demonstrated marginal improvements in latrine infrastructure which in turn influenced latrine usage [14]. In a cross-sectional study in Ethiopia, clean latrine and latrine with a protected door were considerably associated with regular latrine usage [9]. Thus, improvements in latrine infrastructure determined consistent latrine usage among rural masses. Presence of adequate lighting inside the latrine influenced latrine usage among rural households and this paves a way to use the latrines regularly both in the day and night time. Similarly, in Orissa, a longitudinal study found that absence of light inside a latrine was one of the reasons for inconsistent latrine usage [12]. Distinctively, a few villagers used the wastes from their latrines as manure for their crops. In Orissa, three years after TSC implementation, outcomes showed that a functional twin pit latrine was associated with increased latrine utilization when compared to open pit latrine [12].

This community-based study successfully captured the psychological, socio-cultural and structural factors influencing latrine ownership and utilization in rural areas. We covered an adequate representative rural household and the questionnaire was developed in alignment with the standard guidelines given by Swachh Bharat Mission. Non-response rate was minimal owing to good rapport development through our existing community-based services in the study villages. Misclassification bias on account of self-reported latrine ownership was minimized by employing triangulation in data collection where direct observation of the latrine facility using a check-list was done along with the survey. As the field level surveys were undertaken by the Principal Investigator in supervision of an independent faculty, Interviewer's bias was minimal. Nevertheless, the present study also had certain limitations which were undeniable. Social desirability bias in the self-reported latrine usage would occur despite having a good rapport with the villagers. On account of feasibility, as we relied on the female head of the family for obtaining information regarding the latrine usage of other family members, misclassification bias might occur in the present study. Unlike other sanitation intervention studies, the present study did not emphasize on the health outcomes related to latrine usage.

## Conclusion:

In spite of government subsidies for latrine construction through Total Sanitation Campaign (TSC) and Swachh Bharat Mission, the levels of latrine utilization were lower than the levels of latrine ownership. Hence, it is crucial to explore the psychological, socio-cultural and structural factors facilitating latrine construction and consistent utilization. Acceleration of community sensitization and mobilization also plays a pivotal role in sustainable sanitation. Therefore, a multi-pronged strategy employing context-specific behaviour change communication along with Government's financial support would improve both latrine construction and utilization in resource poor settings.

## Financial support and sponsorship:

NIL

## Figures and Tables

**Table 1:** Socio-demographic characteristics of the households in the study villages [N=422]

**Socio-demographic Characteristics**	**N (%)**
Age of the respondents in years (female head of the family)	
20-60	382 (90.5)
>60	40 (9.5)
Occupation of the head of the family	
Professional/ Semi-professional	3 (0.7)
Clerical/ shop-owner/ farm-owner	44 (10.4)
Skilled worker/ Semi-skilled worker	116 (27.5)
Unskilled worker	198 (46.9)
Housewife	40 (9.5)
Retired/ Unemployed	21 (5.0)
Education of the head of the family	
Illiterate	138 (32.7)
Primary/ Middle/ High school	210 (49.8)
Higher secondary	53 (12.6)
Graduate	21 (5.0)
Religion	
Hindu	417 (98.8)
Christian	2 (0.5)
Muslim	3 (0.7)
Caste	
Backward caste	123 (29.2)
Scheduled caste	299 (70.8)
Type of house	
Pucca	158 (37.4)
Semi-pucca	153 (36.3)
Kutcha	111 (26.3)
Type of family	
Nuclear	299 (70.8)
Joint	67 (15.9)
Three-Generation	56 (13.3)
Total number of family members	
01-May	340 (80.6)
06-Oct	79 (18.7)
> 10	3 (0.7)
Monthly income of the family	
Above poverty line	258 (61.1)
Below poverty line	164 (38.9)

**Table 2:** Households' self-perceived reasons for having a latrine [N=231] (multiple options)

**Categories**	**Reasons for having a latrine**	**n (%)**
Psychological factors	For convenience	69 (29.9)
	Habitual	9 (3.9)
	Latrine ensured privacy	43 (18.6)
	Latrine provided safety and protection	47 (20.3)
	Cleanliness and hygiene	8 (3.5)
	Prevention of diseases and snake bites	13 (5.6)
	For emergency purpose	39 (16.9)
	Not sure of having it	9 (3.9)
Socio-cultural factors	Dignity and Status	45 (19.5)
	For women, adolescents and guests	6 (2.6)
Structural factors	Reduced space for Open Defecation	3 (1.3)
	Government built for free	19 (8.2)

**Table 3:** Households' self-perceived reasons for using the owned latrine [N=108] (multiple options)

**Categories**	**Reasons for using the owned latrine**	**n (%)**
Psychological factors	Latrine usage was convenient	34 (31.5)
	Latrine usage ensured privacy	31 (28.7)
	Fear of diseases and snake bites	34 (31.5)
	Latrine usage provided safety and protection	9 (8.3)
	Latrine usage saved time	14 (13.0)
	Open defecation was disgusting	18 (16.7)
	For emergency purposes	2 (1.8)
Sociocultural factors	Dignity and Status	21 (19.4)
	Education and occupation related	13 (12.0)
Structural factors	Proper functioning latrine	64 (59.3)
	Water source nearby	52 (48.1)
	Latrine built near the house	38 (35.2)
	Adequate lighting in the latrine	26 (24.1)
	Manure for crops	10 (9.3)

## References

[1] Coffey D (2017). Econ Political Wkly..

[2] Juran L (2019). Environ Manage..

[3] Ravindra K (2019). Environ Sci Pollut Res Int..

[4] Behera MR (2021). J Educ Health Promot..

[5] Sheethal MP, Shashikantha SK (2016). Int J Community Med Public Health..

[6] Daniel S (2017). Int J Community Med Public Health.

[7] Anuradha R (2017). Indian J Community Med.

[8] Ashraf S (2021). JMIR Res Protoc..

[9] Alemu F (2018). BMC Public Health.

[10] Routray P (2015). BMC Public Health.

[11] Boisson S (2014). India. BMC Public Health.

[12] Barnard S (2013). PLoS One.

[13] Surya AV (2017). Procedia Comput Sci..

[14] Pickering AJ (2015). Lancet Glob Health..

